# Playgrounds Location and Patterns of Use

**DOI:** 10.21203/rs.3.rs-2697497/v1

**Published:** 2023-03-21

**Authors:** Deborah Young, Thomas L McKenzie, Sarah Eng, Meghan Talarowski, Bing Han, Stephanie Williamson, Emily Galfond, Deborah A Cohen

**Affiliations:** KPSC: Kaiser Permanente Southern California; San Diego State University; Kaiser Permanente Southern California; Studio Ludo; Kaiser Permanente Southern California; RAND Corporation; Studio Ludo; Kaiser Permanente Southern California

**Keywords:** Public health, playgrounds, residential location, non-motorized travel, neighborhoods, survey

## Abstract

Playgrounds have features that benefit visitors, including opportunities to engage in outdoor physical activity. We surveyed 1350 adults visiting 60 playgrounds across the U.S. in Summer 2021 to determine if distance to the playground from their residence was associated with weekly visit frequency, length of stay, and transportation mode to the site. About 2/3 of respondents living within ½ mile from the playground reported visiting it at least once per week compared with 14.1% of respondents living more than a mile away. Of respondents living within ¼ mile of playgrounds, 75.6% reported walking or biking there. After controlling for socio-demographics, respondents living within ½ mile of the playground had 5.1 times the odds (95% CI: 3.68, 7.04) of visiting the playground at least once per week compared with those living further away. Respondents walking or biking to the playground had 6.1 times the odds (95% CI: 4.23, 8.82) of visiting the playground at least once per week compared with respondents arriving via motorized transport. For public health purposes, city planners and designers should consider locating playgrounds ½ mile from all residences. Distance is likely the most important factor associated with playground use.

## Introduction

Playgrounds are built environments that provide features offering multiple health-related benefits to visitors. They provide venues for youth and adult physical activity,[1] opportunities to develop fine and gross motor skills and foster the development of the vestibular system,[2] create new collaborations and friendships,[3, 4] and spark creativity and problem-solving through brain development.[4] Additionally, time spent outdoors calms the stress response,[4, 5] can help with emotional regulation,[4, 6] supports improved immune system health,[7] and improves eye function.[8]

The placement of playgrounds is associated with frequency of use. Molina-Garcia et al found that children living in neighborhoods with more playgrounds acquired more physical activity than children in neighborhoods with fewer playgrounds.[9] Others have noted similar results.[10]

National guidelines recommend that children obtain 60 minutes of moderate-to-vigorous physical activity per day.[11] Further, the 2016 National Physical Activity Plan states that playgrounds are critical sites to support active recreation.[11]

In the National Study of Playgrounds, we assessed how design and specific playground features was associated with the number of users and time spent in the playgrounds. After accounting for factors like neighborhood population density and percent of households in poverty, we found that specific innovative playground features accounted for approximately 43% more use.[12]

However, physical activity associated with playgrounds is not strictly dependent on time spent in the playground but is also associated with how people get to the playgrounds. Non-motorized travel is a way for adults and youths to obtain physical activity. When playgrounds are located near residential areas, it is possible to walk or bike to enjoy their facilities. Meanwhile, only 20% of homes in the United States are located within a ½ mile of a park.[4]

We examined the association of distance from home and transportation modality with the frequency and duration of playground use across 60 playgrounds in 10 US cities that were a part of the National Study of Playgrounds. We determined how distance from home to playgrounds was associated with use and active transport.

## Materials And Methods

The National Study of Playgrounds was designed to assess the impact of non-schoolyard based playground design on physical activity.[12] Playgrounds with innovative designs (n = 3 per city) were compared to matched traditional post-and-platform playgrounds (n = 3 per city) in the metropolitan areas of Boston, Chicago, Cincinnati, Denver, Houston, Los Angeles, Memphis, New York, San Francisco, and Seattle (i.e., 60 total playgrounds). The playgrounds were matched by socio-economic aspects of the surrounding census tract, including household poverty level and racial/ethnic demographics. As no personal identifying information was gathered, the study was determined by the RAND Institutional Review Board to be exempt from Human Subjects Review.

To understand playground utilization, we created a two-page self-administered questionnaire that was completed by adult playground visitors. The instrument included questions about adult and child(ren) demographic information, transportation to the playground, approximate distance from home, typical frequency of using the playground, reasons for choosing the playground, favorite playground features, and the perceived safety and maintenance of the playground. The questionnaire was administered most frequently in English, but Spanish questionnaires were available.

For this report, we focused on the following questions: 1) How did you get to this playground today (response options: on foot, non-motorized vehicle, motor vehicle, public transport, other)? 2) How often do you usually come to this playground (first time, 5–7 times per week, 2–4 times per week, once per week, 2–3 times per month, several times per year, a few times per year)? 3) On a typical day when you come to the playground, how long do you stay (0–30 minutes, 31–60 minutes, more than 1 hour but less than 2 hours, 2–3 hours, more than 3 hours)? Respondents marked how far the playground was from their residence on a local map and responses were coded as 0-.25 mile, .25-.50 mile, .50 – 1 mile, and more than 1 mile.

Data collectors were trained in all aspects of the National Study of Playgrounds protocol and they visited playgrounds to administer the questionnaire on Wednesdays, Fridays, Saturdays, and Sundays during a single week in Summer, 2021. They approached people in the playground who appeared to be older than 18 years of age. After introducing themselves and explaining they were part of a national study funded by the National Institutes of Health, they asked potential responders if they were willing to complete a short survey, after which they would receive a $5 gift card. No name, address, or identifying information was obtained. The goal was to collect at least 20 completed questionnaires per playground over the course of the week.

## Analysis

Descriptive statistics were used to summarize survey responses by distance from home and mode of transportation to playground (condensed into walk/bike transportation and other transportation modes). Comparisons across groups for categorical and continuous variables were done using Chi-square test and Kruskal-Wallis one-way analysis of variance, respectively.

Frequency of visits and duration of stay were modeled using logistic regression, in which visiting the playground once per week or more and staying more than 1 hour were the outcomes of interest. Frequency of visits and duration of stay variables were highly correlated variables, so the models were run separately with the same covariates. Regression models were adjusted for gender, age, marital status, and relationship to child (married parent, non-married parent, other relationship), education status, race/ethnicity, playground type, and city.

The significance level for descriptive statistics and logistic regression models was set to 0.05. All analyses were conducted using SAS version 9.4 (SAS Institute, Cary, NC).

## Results

Across the 60 playgrounds 1,365 surveys were completed (76.6% response of invites). Of these, complete data were missing for 15 respondents, leaving an analytic data set of 1,350 persons. Most respondents were women (69.9%) and respondents reported a mean age of 38.5 ± 10.9 (Table 1). Most reported they were White (50.6%), with 16.4% reporting being Hispanic, 10.3% Black, 10.6% Asian/Pacific Islander, 0.3% American Indian/Alaska Native, and 11.9% indicating multiple races/ethnicities. The majority (59.6%) reported being employed full-time and over two-thirds reported having a bachelor’s degree or higher.

There were few meaningful demographic differences for reported distance of the playground from one’s residence, although respondents living within ½ mile or ¼ mile from the playgrounds were more likely to be of Hispanic ethnicity or Black race, respectively. There was also a tendency for respondents living closer to the playgrounds to report having a lower educational level, being single, and less likely to be the parent of the child(ren) they brought to the playground. (Table 1). Most reported bringing an average of 2.0 ± 1.80 children with them on the day they completed the survey.

Reported distance from one’s residence was associated with the frequency of playground visits. About 2/3 of respondents living within ¼ mile or ½ mile from the playground reported visiting the playground at least once per week (66.1% and 63.2%, respectively) compared with 14.1% of respondents living more than a mile away. Living closer was associated with shorter visits than living further away. Respondents living close to the playground most often identified convenience as the reasons for visiting that particular playground (Table 2). Reporting that there were no other playgrounds nearby and that friends and other children used that particular playground were significantly endorsed more often by respondents living near the playground than respondents living further away. Irrespective of distance from home, 95% perceived the playground were either “safe” or “very safe.” (data not shown).

A total of 524 respondents (39%) reported they had walked or biked to the playground. As presented in Table 2, walking or biking to playgrounds was more prevalent for respondents living closer to the playground. For respondents living within ¼ mile of playgrounds, 75.6% reported arriving by non-motorized transportation whereas only 8.6% of those living more than 1 mile away reported that transportation mode. Table 3 displays results indicating that those who walked or biked were likely to visit playgrounds more often, although their visits were shorter than those using a motorized vehicle. Almost 2/3 of respondents (64.2%) who walked or biked reported visiting the playground at least once per week compared with 21.7% of those who arrived by vehicle. Nearly one-half of respondents (49.3%) who walked or biked reported staying more than one hour, compared to 58% of those arriving by vehicle.

Tables 4 and 5 present results of the logistic regressions with distance from one’s residence (Table 4) and travel mode (Table 5) as the main exposures and the outcomes of frequency of playground visits and duration of playground visits. After controlling for socio-demographics and playground type, people who reported living within ½ mile of the playground were 5.06 times (95% CI: 3.68, 7.04) more likely to visit the playground at least once per week compared with respondents living further away (Table 4). Those living closer were less likely to report staying an hour or longer (OR: 0.64; 95% CI: 0.47, 0.86) compared to people living at least ½ mile from the playground. Respondents reporting walking or biking to the playground were 6.12 times (95% CI: 4.23, 8.82) more likely to visit the playground at least once per week compared with those arriving via motorized transport. Respondents who walked or bicycled were 37% less likely to stay longer than one hour (OR: 0.63; 95% CI: 0.47, 0.83) compared with those arriving via motorized transport.

## Discussion

Results of this national study provide strong evidence that playground location is associated with the frequency of playground use. When playgrounds are convenient, people visit them more frequently and use non-motorized transportation to get there. While there are many considerations when selecting playground sites, proximity to residential areas is of utmost importance. Ensuring that safe sidewalks and bike lanes are conveniently located may enhance non-motorized travel to playgrounds.

Residents living within ½ mile of a playground were four times more likely to visit it at least once per week. Veitch and colleagues found that going to parks as a family at least weekly was associated with more frequent play in a park or playground compared with families who visited less regularly.[13] Only 20% of homes in the U.S. are located within ½ mile of a park.[4] In contrast, in a study of over 9,000 individuals surveyed in eight Latin American countries, 49% reported accessibility to parks and 82% reported access to playgrounds (accessibility defined as within a 20-min walking distance).[14] City planners, urban designers, and parks departments should consider the importance of playground locations, as well as active transport networks, when creating new or reconfiguring existing neighborhoods. Siting playgrounds within ¼ to ½ mile of every resident should be a goal for all major cities.

Respondents who walked/biked to playgrounds or lived within ½ mile of them reported having shorter visits than those who drove or lived further away. While we did not query why people might stay longer at playgrounds, many of the playgrounds that were closer to residential areas were smaller and had fewer amenities than others and may not have supported as long of a stay time. Additionally, some of the 60 playgrounds chosen for this study were situated within parks, and people may have left the playground to use other park facilities. Nonetheless, the additional frequency of playground visits may compensate for shorter visits and influence the time to engage in play and physical activity.

Play is an essential building block for youth development. Outdoor play, which often occurs in playgrounds, stimulates problem-solving and creative thinking, provides opportunities for social interactions, and can improve emotional well-being and mood.[15] We and others[13, 16] found that when playgrounds are available in neighborhoods there is a greater likelihood of children playing outdoors. More children could reap the benefits of outdoor play if more playgrounds were conveniently located.

Another strength of playgrounds being located near residential areas are the opportunities for walking and biking to them. Proximity thus provides additional opportunities for children and adults to meet the national guidelines for physical activity.[11] For adults, moderate-intensity physical activity can be quantified as walking 1 mile at a pace of 2.5–4.0 mph,[11] or roughly in 15–25 minutes. Thus, an adult living within ½ mile of a playground could obtain up to 25 minutes of the minimum of 150 minutes/week recommendation for each visit.

The study had limitations. Surveys were conducted only during the summer, and respondents may have answered differently during other seasons. Not all adults agreed to complete the survey, so the respondents may not be representative of all adults visiting the playgrounds. How often respondents reported their frequency of visits and typical duration of stay were self-reported and could not be verified. All 60 playgrounds in the study were either built or renovated in the previous 10 years and, therefore, are not representative of all playgrounds. Nonetheless, there were many study strengths. Trained assessors visited 60 playgrounds across 10 cities in the U.S., thus being able to provide national rather than only city or regional information. Additionally, surveys were completed by more than 1,300 adults who were visiting the playgrounds.

## Conclusion

The location of playgrounds near residential areas was associated with greater frequency of their use and the use of active (i.e., non-motorized transportation) to get there. City planners and urban designers should consider finding spaces for playgrounds that are ideally within ¼ to ½ mile of residential location. This will result in more opportunities for physical activity for children and adults and likely contribute to public health.

## Figures and Tables

**Table 1:** Participant characteristics of N = 1350 survey respondents by distance from residence to playground, U.S. adults, 2021.

	0 – .25 mile	.25 – .50 mile	.50 – 1 mile	More than 1 mile	Total	P-value
	(N = 385)	(N = 114)	(N = 179)	(N = 672)	(N = 1350)	
**Gender** n (%)						0.303^1^
Female	286 (74.3%)	83 (72.8%)	120 (67.0%)	454 (67.6%)	943 (69.9%)	
Male	98 (25.5%)	30 (26.3%)	58 (32.4%)	213 (31.7%)	399 (29.6%)	
Non-Binary	1 (0.3%)	1 (0.9%)	1 (0.6%)	5 (0.7%)	8 (0.6%)	
**Age, Mean (SD)**	39.1 (12.26)	37.8 (10.21)	37.3 (9.88)	38.6 (10.32)	38.5 (10.85)	0.670^1^
**Race or ethnicity**, n (%)						< 0.001^2^
Hispanic/Latino	81 (21.0%)	28 (24.6%)	28 (15.6%)	84 (12.5%)	221 (16.4%)	
White	160 (41.6%)	54 (47.4%)	89 (49.7%)	380 (56.5%)	683 (50.6%)	
Black	61 (15.8%)	8 (7.0%)	12 (6.7%)	58 (8.6%)	139 (10.3%)	
Asian/Pacific Islander	38 (9.9%)	9 (7.9%)	24 (13.4%)	72 (10.7%)	143 (10.6%)	
American Indian/Alaska Native	1 (0.3%)	0 (0.0%)	1 (0.6%)	2 (0.3%)	4 (0.3%)	
Other	44 (11.4%)	15 (13.2%)	25 (14.0%)	76 (11.3%)	160 (11.9%)	
**Employment status**, n (%)						0.613^1^
Working full time	218 (58.4%)	68 (59.6%)	100 (59.2%)	394 (60.3%)	780 (59.6%)	
Working part time	52 (13.9%)	15 (13.2%)	26 (15.4%)	83 (12.7%)	176 (13.4%)	
Stay-at-home caregiver	33 (8.8%)	14 (12.3%)	21 (12.4%)	80 (12.3%)	148 (11.3%)	
Other (Unemployed, Retired, Disabled, Student)	70 (18.8%)	17 (14.9%)	22 (13.0%)	96 (14.7%)	205 (15.7%)	
**Educational level**, n (%)						0.001^1^
High school graduate or less	60 (15.6%)	14 (12.3%)	13 (7.3%)	47 (7.0%)	139 (10.3%)	
Some college or AA degree	88 (22.9%)	16 (14.0%)	0 (0.0%)	3 (0.4%)	236 (17.5%)	
Bachelor’s Degree	109 (28.3%)	43 (37.7%)	0 (0.0%)	7 (1.0%)	455 (33.7%)	
Master’s Degree or higher	121 (31.4%)	40 (35.1%)	67 (37.4%)	236 (35.1%)	492 (36.4%)	
Missing	7 (1.8%)	1 (0.9%)	13 (7.3%)	42 (6.3%)	28 (2.1%)	
**Current marital status**, n (%)						< 0.001^1^
Single	112 (29.8%)	31 (27.2%)	51 (29.1%)	135 (20.4%)	329 (24.8%)	
Married or domestic partnership	234 (62.2%)	69 (60.5%)	116 (66.3%)	493 (74.5%)	912 (68.7%)	
Other	30 (8.0%)	14 (12.3%)	8 (4.6%)	34 (5.1%)	86 (6.5%)	
**Primary relationship to the child(ren)**, n (%)						< 0.001^1^
Parent	223 (58.7%)	75 (67.0%)	120 (68.6%)	498 (75.5%)	916 (69.0%)	
Babysitter/Nanny	60 (15.8%)	14 (12.5%)	25 (14.3%)	47 (7.1%)	146 (11.0%)	
Other	97 (25.5%)	23 (20.5%)	30 (17.1%)	115 (17.4%)	265 (20.0%)	
**Number of children brought to the playground**, Mean (SD)	2.1 (2.41)	2.0 (1.82)	2.1 (2.24)	1.9 (1.15)	2.0 (1.80)	0.531^1^

1 Chi-Square p-value

2 Kruskal-Wallis p-value

**Table 2:** Transportation mode to playground, frequency, duration, and reason for visiting the playground, U.S. adults, 2021.

	0 – .25 mile	.25 – .50 mile	.50 – 1 mile	More than 1 mile	Total	P-value^1^
	(N = 385)	(N = 114)	(N = 179)	(N = 672)	(N = 1350)	
**Mode of Transport**, n (%)						< .0001
Walked	291 (75.6%)	68 (60.2%)	68 (38.0%)	58 (8.6%)	485 (36.0%)	
Biked or other non-motorized vehicle	6 (1.6%)	5 (4.4%)	9 (5.0%)	19 (2.8%)	39 (2.9%)	
Motorized Vehicle	88 (22.9%)	40 (35.4%)	102 (57.0%)	595 (88.5%)	825 (61.2%)	
**Frequency of playground visits**, n (%)						< .0001
This is the first time	27 (7.0%)	15 (13.2%)	14 (7.8%)	212 (31.8%)	268 (20.0%)	
Occasionally (a few times per year, but less than once per week)	103 (26.9%)	27 (23.7%)	70 (39.1%)	361 (54.1%)	561 (41.8%)	
Once per week or more	253 (66.1%)	72 (63.2%)	95 (53.1%)	94 (14.1%)	514 (38.3%)	
**Duration of playground visits**, n (%)						0.001
0–30 minutes	33 (8.6%)	14 (12.3%)	14 (7.8%)	37 (5.5%)	98 (7.3%)	
31–60 minutes	159 (41.3%)	50 (43.9%)	74 (41.3%)	233 (34.7%)	516 (38.2%)	
More than 1hr, less than 2hr	87 (22.6%)	32 (28.1%)	57 (31.8%)	227 (33.8%)	403 (29.9%)	
2–3hrs	82 (21.3%)	13 (11.4%)	29 (16.2%)	136 (20.2%)	260 (19.3%)	
More than 3hrs	7 (1.8%)	2 (1.8%)	2 (1.1%)	19 (2.8%)	30 (2.2%)	
**Why did you choose this playground over others nearby? n (%)**						
There are no other playgrounds nearby	31 (8.1%)	8 (7.0%)	6 (3.4%)	24 (3.6%)	69 (5.1%)	0.007
It is conveniently located	296 (76.9%)	91 (79.8%)	128 (71.5%)	225 (33.5%)	740 (54.8%)	< .0001
Facilities/equipment suitable for children	208 (54.0%)	73 (64.0%)	106 (59.2%)	386 (57.4%)	773 (57.3%)	0.254
Facilities/equipment suitable for adults	49 (12.7%)	19 (16.7%)	15 (8.4%)	119 (17.7%)	202 (15.0%)	0.008
Friends and their children use the playground too	91 (23.6%)	34 (29.8%)	42 (23.5%)	129 (19.2%)	296 (21.9%)	0.046
Tourist attraction or destination playground	21 (5.5%)	7 (6.1%)	12 (6.7%)	172 (25.6%)	212 (15.7%)	< 0.001
Attend park-sponsored program or event	9 (2.3%)	2 (1.8%)	6 (3.4%)	14 (2.1%)	31 (2.3%)	0.759
Attend social events of friends or family	13 (3.4%)	7 (6.1%)	8 (4.5%)	34 (5.1%)	62 (4.6%)	0.520
None of the above, n (%)	9 (2.3%)	2 (1.8%)	10 (5.6%)	52 (7.7%)	73 (5.4%)	0.001

1 Chi-Square p-value

**Table 3:** Reported distance, frequency, and duration of playground visits by transportation mode, U.S. adults, 2021.

	Walked/Biked	Other Transportation	Total	P-value
	(N = 524)	(N = 826)	(N = 1350)	
**Distance from home**, n (%)				< 0.0001^1^
0–.25 mile	297 (56.7%)	88 (10.7%)	385 (28.5%)	
.25–.50	73 (13.9%)	41 (5.0%)	114 (8.4%)	
.50 – 1 mile	77 (14.7%)	102 (12.3%)	179 (13.3%)	
More than 1-mile	77 (14.7%)	595 (72.0%)	672 (49.8%)	
**Frequency of visits**, n (%)				< 0.0001
This is the first time	48 (9.2%)	220 (26.7%)	268 (19.9%)	
5–7 times per week	100 (19.2%)	15 (1.8%)	115 (8.6%)	
2–4 times per week	172 (33.0%)	100 (12.2%)	272 (20.2%)	
Once per week	64 (12.3%)	63 (7.7%)	127 (9.4%)	
2–3 times per month	77 (14.8%)	138 (16.8%)	215 (16.0%)	
Several times per year	37 (7.1%)	133 (16.2%)	170 (12.6%)	
A few times per year	24 (4.6%)	152 (18.5%)	176 (13.1%)	
**Duration of visits**, n (%)				0.00091
0–30 minutes	55 (10.7%)	43 (5.3%)	98 (7.4%)	
31–60 minutes	210 (40.9%)	306 (37.5%)	516 (38.9%)	
More than 1hr, less than 2hr	134 (26.1%)	269 (33.0%)	403 (30.3%)	
2–3 hrs	95 (18.5%)	165 (20.2%)	260 (19.6%)	
More than 3hrs	9 (1.8%)	21 (2.6%)	30 (2.3%)	

1 Chi-Square p-value

**Table 4:** Logistic regression analysis modeling frequency playground visits (once a week or more) and duration of playground visit (more than 1 hour) by playground distance from home, U.S. adults, 2021.

	Frequency of Playground Visits (At least once per week)		Duration of Playground Visits (More than one hour)	
	Odds Ratio	95% CI	Odds Ratio	95% CI
Playground distance				
Less than ½ mile from home	**5.09**	**3.68, 7.04**	**0.64**	**0.47, 0.86**
½ mile or greater from home	1.0	-	1.0	-
Gender				
Female	0.90	0.66, 1.23	1.26	1.02, 1.57
Non-binary	0.57	0.10, 3.43	1.29	0.30, 5.54
Male	1.0	-	1.0	-
Respondent age	**0.98**	**0.96, 0.99**	**1.01**	**1.00, 1.02**
Relationship to child				
Non-married parent	**1.45**	**1.07, 1.95**	**1.13**	**0.87, 1.48**
Other relationship to child	1.61	0.98, 2.65	1.05	0.67, 1.63
Married parent	1.0	-	1.0	-
Education				
High school graduate or less	1.45	0.92, 2.29	1.27	0.78, 2.08
Some college or Associate degree	1.03	0.68, 1.55	1.06	0.71, 1.58
College graduate	1.07	0.77, 1.49	1.07	0.78, 1.48
Post- bachelor’s degree	1.0	-	1.0	-
Race/ethnicity				
Asian/Pacific Islander	1.20	0.85, 1.70	1.17	0.78, 1.74
Black	**0.67**	**0.46, 1.00**	0.85	0.54, 1.36
Hispanic/Latino	1.21	0.81, 1.79	1.08	0.69, 1.70
Other	1.02	0.66, 1.58	**1.73**	**1.10, 2.73**
White	1.0	-	1.0	-
Playground type				
Innovative	**0.65**	**0.50, 0.86**	**1.49**	**1.03, 2.15**
Traditional	-	-	-	-

**Table 5:** Logistic regression analysis modeling frequency playground visits (once a week or more) and duration of playground visit (more than 1 hour) by mode of transportation to the playground, U.S. adults, 2021.

	Frequency of Playground Visits (At least once per week)		Duration of Playground Visits (More than one hour)	
	Odds Ratio	95% CI	Odds Ratio	95% CI
Mode of travel to playground				
Walked or bicycled	**6.11**	**4.23, 8.82**	**0.63**	**0.47. 0.83**
Motorized transport	1.0	-	1.0	-
Gender				
Female	0.97	0.70, 1.33	1.25	1.01, 1.55
Non-binary	0.64	0.16, 2.61	1.23	0.26, 5.78
Male	1.0	-	1.0	-
Respondent age	**0.98**	**0.97, 1.00**	1.01	0.99, 1.02
Relationship to child				
Non-married parent	1.34	0.95, 1.89	1.15	0.88, 1.51
Other relationship to child	1.34	0.81, 2.22	1.09	0.71, 1.67
Married parent	1.0	-	1.0	-
Education				
High school graduate or less	**1.73**	**1.12, 2.65**	1.22	0.74, 1.99
Some college or Associate degree	1.29	0.87, 1.92	1.00	0.67, 1.50
College graduate	1.02	0.73, 1.42	1.08	0.79, 1.48
Post- bachelor’s degree	1.0	-	1.0	-
Race/ethnicity				
Asian/Pacific Islander	1.27	0.88, 1.84	1.16	0.78, 1.73
Black	0.90	0.60, 1.34	0.80	0.50, 1.28
Hispanic/Latino	**1.60**	**1.05, 2.42**	1.10	0.65, 1.59
Other	1.15	0.76, 1.75	**1.69**	**1.06, 2.70**
White	1.0	-	1.0	-
Playground type				
Innovative	**0.53**	**0.41, 0.69**	**1.57**	**1.08, 2.28**
Traditional	-	-	-	-
